# Luminescence nanothermometry with alkyl-capped silicon nanoparticles dispersed in nonpolar liquids

**DOI:** 10.1186/1556-276X-9-94

**Published:** 2014-02-24

**Authors:** Hamza Hajjaji, Sergey Alekseev, Gérard Guillot, Nicholas P Blanchard, Virginie Monnier, Yann Chevolot, Georges Brémond, Michel Querry, David Philippon, Philippe Vergne, Jean Marie Bluet

**Affiliations:** 1 Université de Lyon, CNRS, UMR 5270, INSA-Lyon, INL, Villeurbanne 69621, France; 2 Chemistry Faculty, Kiev National Taras Shevchenko University, Kiev 01601, Ukraine; 3 Université de Lyon, CNRS, UMR 5306, Université Claude Bernard Lyon1, ILM, Villeurbanne 69622, France; 4 Université de Lyon, CNRS, UMR 5270, EC-Lyon, INL, Ecully 69134, France; 5 Université de Lyon, CNRS, UMR5259, INSA-Lyon, LaMCoS, Villeurbanne 69621, France

**Keywords:** Silicon nanoparticles, Photoluminescence, Functionalization, Low-polarity liquids, Temperature sensing

## Abstract

Silicon nanoparticles (Si NPs) with a diameter size ranging from 4 to 8 nm were successfully fabricated. They exhibit a visible photoluminescence (PL) due to the quantum confinement effect. Chemical functionalization of these Si NPs with alkyl groups allowed to homogeneously disperse them in nonpolar liquids (NPLs). In comparison to most of literature results for Si NPs, an important PL peak position variation with temperature (almost 1 meV/K) was obtained from 303 to 390 K. The influence of the liquid viscosity on the peak positions is also presented. These variations are discussed considering energy transfer between nanoparticles. The high PL thermal sensitivity of the alkyl-capped Si NPs paves the way for their future application as nanothermometers.

## Background

Silicon nanoparticles (Si NPs) or ‘quantum dots’ (QDs) are widely investigated by the scientific community because of their interesting optical and electronic properties which differ from those of the bulk Si and, consequently, their potential use in several applications ranging from nanoelectronic to optoelectronic and photovoltaic devices or biological imaging [1-3]. At nanometer scale, Si NPs in colloidal form exhibit visible photoluminescence (PL) with a high quantum yield because of the confinement effect which partly overcomes the indirect band gap and which can be tuned by the NP size [4-6]. However, PL from oxidized Si QDs has low radiative rates and is not spectrally tunable [7]. H-terminated Si QDs have spectrally tunable PL but also low radiative rates and are chemically unstable and easily oxidable [7,8]. Dedicated surface engineering such as alkyl chains by organic capping involving a carbon surface termination has led recently to bright luminescent Si NPs [9-13]. These NPs have stable surface passivation due to the strong covalent Si-C bond preventing photo-oxidation and aggregation in solution [14]. This allows also versatile (bio)functionalization [15]. They are nontoxic [16] and show bright photo-stable blue-green PL with fast decay for 2- to 3-nm size [17,18].

In this study, our goal is to use Si NPs as nanothermometers in nonpolar liquids (NPLs). The main application is temperature measurements (in the range of 0°C to 120°C) in lubricant for tribological studies of mechanical contacts. As dispersion in nonpolar liquids (alkane or alkenes for example) is required, we use alkyl surface termination. Nanothermometers based on II-VI semiconductor QDs have been reported [19,20]. In spite of some disadvantages of the II-VI materials relative to Si such as toxicity, scarcity of material resource, and instability, only few published works report on the use of Si NPs as nanothermometers [21].

We show an important PL peak position variation with temperature for Si NP colloids (approximately 1 meV/K). The investigation of Si NP luminescence property variation both with temperature and liquid medium viscosity gives an original demonstration of the exchange energy transfer (EET) importance in Si NP colloids.

## Methods

Electrochemical anodic etching of p-type 10-Ω cm (100)-oriented Si wafer has been used for the preparation of nano-Si powder. Silicon substrate was etched in a solution containing 1:1 volume mixture of 48% hydrofluoric acid (HF) and anhydrous ethanol. The anodization was performed in a Teflon cell with a copper electrode as a backside contact. The counter electrode was made of platinum. Anodic current density was 45 mA/cm^2^ and etching time was 50 min. A permanent stirring of the etching solution was applied in order to evacuate hydrogen bubbles formed during the etching process. After the etching, a highly porous network constituted of numerous interconnected nanocrystals was formed. The sample was washed with anhydrous ethanol several times, dried in ambient air, and then scratched out from the wafer.

Thermal hydrosilylation approach was used for the grafting of octadecyl groups (-C_18_H_37_) onto the surface of the Si NPs. As exposition of highly porous Si to ambient air results in its oxidation, the surface oxide was removed using a 5% solution of HF in EtOH just before the hydrosilylation. The residues of acid were washed out by anhydrous EtOH (under centrifugation). The oxide-free porous Si powder (covered by SiH_x_) was transferred in a glass test tube with septum cup and dried under vacuum in order to remove excess EtOH. Then, 1.5 mL of neat 1-octadecene was added, and the reaction mixture was stirred under nitrogen atmosphere at 150°C for 16 h. At the end of this step, the surface of Si NPs is mainly covered by alkyl chains due to the hydrosilylation reaction. To work up the reaction mixture, it was cooled to room temperature; the precipitate was settled by centrifugation (10 min at 1,000 × *g*) and washed three times with *n*-pentane. Then, the precipitate was sonicated for 30 min in *n*-pentane, and the supernatant of the centrifugation of the resulted slurry was taken. Drying of the supernatant in ambient air resulted in approximately 10 mg of waxy brown residue, which is easily redispersible in NPLs and which was used for further PL studies.

Transmission electron microscopy (TEM) was used to characterize the morphology and the size distribution of the Si NPs. A droplet of the colloidal solution was deposited on a Cu grid covered by an amorphous carbon film (ultrathin carbon <3 nm). After solvent evaporation, the observation was done using a Topcon EM-002B high-resolution transmission electron microscope operating at 200 kV (Topcon Corporation, Tokyo, Japan).

Particles size distribution of the final solution was also measured by dynamic light scattering (DLS) technique using a Zetasizer Nano Series instrument from Malvern Instruments Ltd. (Worcestershire, UK).

Transmittance Fourier transform infrared (FTIR) spectra of Si NPs were recorded in between KBr pellets in the 400- to 4,000-cm^−1^ spectral range at 300 K using a Bruker Vertex 80 spectrometer (Bruker Optik GmbH, Ettlingen, Germany) before and after the functionalization step.

The PL steady state measurements of Si NPs were performed by means of a FLS920 Series fluorescence spectrometer from Edinburgh Instruments (Livingston, UK). A 450-W Xe900 continuous xenon arc lamp with optimal spectral range extending from 250 to 1,000 nm was used as the excitation source. Excitation and emission beam lights are dispersed by a single-grating monochromator blazed at 500 nm. All spectra were corrected automatically by the transfer function of the instrument. The temperature was varied using a Peltier module between 303 and 383 K. Hellma UV transparent quartz cuvettes (Hellma GmbH & Co. KG, Müllheim, Germany) were used with typical liquid volumes of 1.5 mL.

## Results and discussion

**Figure 1:**
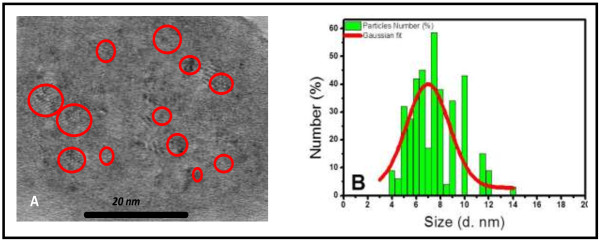
**Transmission electron microscopy image and DLS measurement. (A)** TEM image of Si powder initially suspended in ethanol and deposited on a graphite grid. **(B)** DLS of functionalized Si NPs dispersed in squalane.

**Figure 2:**
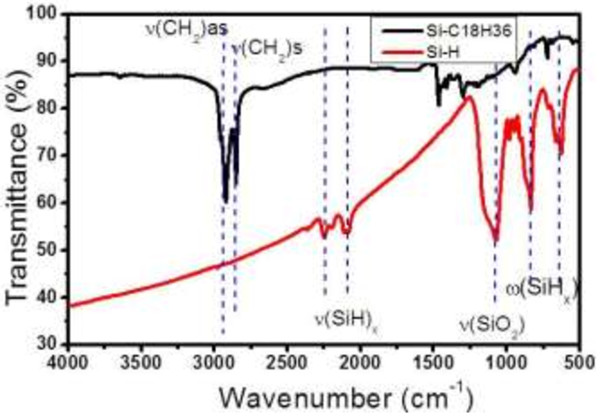
**FTIR analysis of Si NPs before and after functionalization.** Si-C_18_H_37_ means Si NPs functionalized by the C_18_H_37_ group (black curve), and Si-H means Si NPs without any chemical modification (red curve).

Figure 1A shows a TEM image of Si powder suspended in ethanol after two size selection steps: (i) ultrasonic bath at 37 kHz for 10 min in order to separate aggregates and (ii) 10 min of centrifugation at 1,500 × *g*. Monocrystalline Si NPs are observed with a lattice space of 0.31 nm corresponding to the Si (111) plane. Their diameter is mainly ranging from 4 to 8 nm with the presence of few smaller and larger NPs. This size distribution has been confirmed on functionalized Si NPs dispersed in squalane by DLS measurement (Figure 1B). We observe an almost monodisperse size distribution centered at 7 nm with a standard deviation of 2 nm. The efficiency of the functionalization step (Si-C_18_H_37_) has been checked by FTIR analysis of Si NPs before and after reaction. As can be deduced from Figure 2, the surface of initial Si NPs is mainly covered by a native oxide layer giving a large characteristic SiO_2_ band (Si-O-Si symmetric and asymmetric stretching mode) centered at 1,100 cm^−1^. Nevertheless, the presence of H at the surface is also clearly evidenced by SiH_x_ waging and rolling modes around 650 cm^−1^, O_y_-SiH_x_ waging around 850 cm^−1^, SiH_x_ stretching modes at 2,090 cm^−1^, and O_y_-SiH_x_ stretching around 2,230 cm^−1^. After the functionalization, (i) the SiO_2_ band is no longer detected which confirms the success of the HF washing step to remove the oxide layer, and (ii) the different Si-H and O-Si-H related bands disappear. At the same time, characteristic bands of *ν*_as_ (CH_3_) at 2,962 cm^−1^, *ν*_as_ (CH_2_) at 2,925 cm^−1^, *ν*_s_ (CH_2_) at 2,853 cm^−1^, and *δ* (CH_2_) at 1,467 cm^−1^ rise. These data prove the efficient replacement of the Si-H and Si-O bonds by the alkyl chains (C_18_H_37_). After this essential step that leads to a good dispersion of the Si NPs in nonpolar liquid, their luminescence properties were studied.

**Figure 3:**
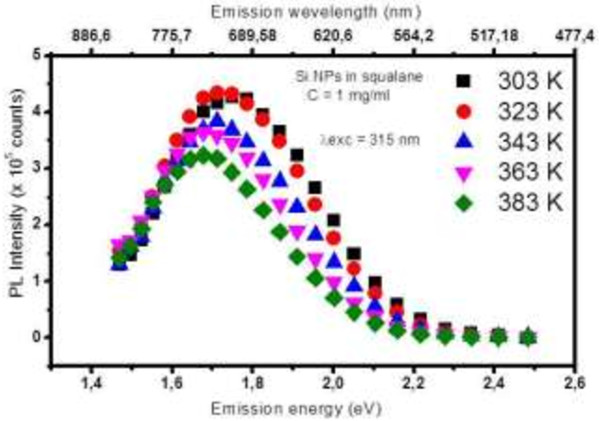
Temperature-dependent fluorescence spectra of Si NP colloidal suspension in squalane with a concentration of 1 mg/mL.

Figure 3 shows temperature-dependent fluorescence spectra of Si NP colloidal suspension in squalane with a concentration C equal to 1 mg/mL. Excitation energy is fixed at the maximum of the excitation spectra (3.94 eV).

**Figure 4:**
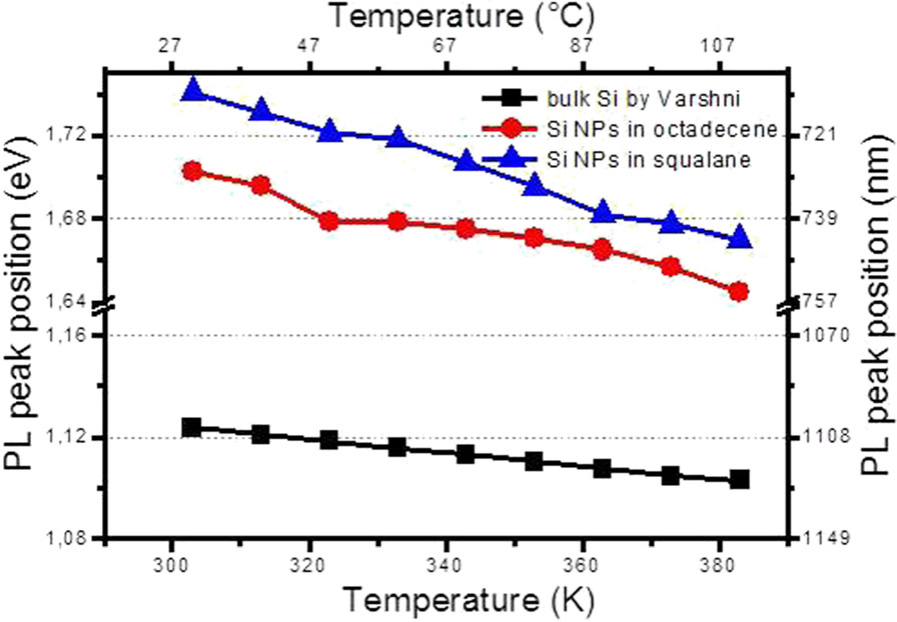
**Temperature dependence and band gap variation.** Temperature dependence of the PL peak position of Si NPs in squalane (blue curve) and in octadecene (red curve), and band gap variation of the bulk Si following the Varshni model (black curve) in the temperature range from 303 to 383 K.

The PL intensity of the Si NPs decreases in the chosen temperature range (from 303 to 383 K). In static conditions, this intensity variation can be used to design a sensitive temperature sensor, but many other parameters can influence the PL intensity in dynamic conditions of a mechanical contact (concentration gradient in the lubricant, pressure variation, nanoparticle flows, etc.). As already published [19], a more intrinsic parameter related to the PL intensity is the PL lifetime. Here we focus on the PL peak position. Clearly, in Figure 3, we can see that due to heating, PL spectra of Si NPs move towards smaller emission energy. Figure 4 describes this evolution of the temperature-dependent PL peak position of Si NPs in squalane and in octadecene. Both are compared to the band gap variation of the bulk Si in the same temperature range obtained from the Varshni model [22]. From our measurements, significant linear red shifts were extracted with a slope equal to −0.63 meV/K (0.28 nm/K) and −0.91 meV/K (0.39 nm/K) in octadecene and squalane, respectively. As evidenced from Figure 4, the temperature dependence of our NP fluorescence energy is much more important than the bulk material band gap variation (three times for Si NPs in octadecene and four times for NPs in squalane). Several experiments have reported on the temperature dependence of PL matrix-embedded (ME) Si NPs [23,24]. They concluded that the blueshift of the PL peak position with decreasing temperature behaves similarly to that of bulk silicon, i.e., the PL blueshift decreases by about 50 meV when the temperature drops from 300 down to 3 K. Near 300 K, the variation is almost linear with a maximal slope below 0.3 meV/K. As reported by Chao et al. [25], upon vacuum ultraviolet excitation of alkylated Si nanocrystallites, intense blue and orange emission bands were found simultaneously. Both peak positions are shifted to longer wavelengths as the temperature increases from 8 K to room temperature: the orange peak position shifts from 600 ± 2 to 630 ± 2 nm. They suggest that this results from the population of localized tail states formed by the disordered potential at the surface [26] due to the surface roughness and variations in surface stoichiometry. A recent study by Kůsová et al. [27] on free-standing (FS) Si nanocrystals obtained from electrochemical anodization has shown a considerably higher blueshift of the emission: 200 meV from 300 down to 4 K with a variation at 300 K of around −1 meV/K which is close to our results for Si NPs in NPLs. Kůsová et al. [27] explained the difference in the shift between FS and ME NPs by the presence of compressive strain in ME NPs which is absent in the case of FS NPs. This explanation is supported by the consideration of a strongly enhanced thermal expansion coefficient for Si NPs (9.10^−6^ K^−1^ instead of 2.10^−6^ K^−1^ for the bulk material). Nevertheless, in another recent work, size-purified plasma-synthesized Si NPs have been studied in the form of pure nanocrystal films and in the form of nanocomposite of Si NPs embedded in polydimethylsiloxane (PDMS) [28]. Strong compressive strain by an oxide matrix cannot be considered in this case. The quantitative deviation of the PL energy *E* with temperature (d*E*/d*T*) for both Si NP samples was found to be the same. A small deviation in comparison with the bulk material is shown in this work with a maximal variation at 300 K of −0.4 meV/K for the smallest NPs (3.2-nm diameter) still far from the −0.9 meV/K obtained in the current work. Furthermore, this deviation is decreasing with the nanoparticle diameter. As our nanoparticle has an average diameter of 7 nm, our results differ from those of the reference [28]. The main difference may lie in the fact that the size distribution is a little scattered which can be at the origin of the important red shift observed when increasing temperature.

The Brownian motion of the NPs in the suspension increases with temperature; at the same time, their mobility also increases as the viscosity of the NPL strongly decreases. This leads to an enhanced probability of energy transfer between NPs in close vicinity. The Förster resonant energy transfer (FRET) of NPs with different sizes strongly depends on the distance *D* between two particles (approximately *D*^−6^) [29]. When the dynamic viscosity of the liquid decreases, it leads to high FRET probability for small NPs (approximately 4 nm in diameter) with larger band gaps toward big NPs (approximately 9 nm in diameter) having smaller band gaps. Thus, the small NPs are optically inactive from the photo-stimulated emission point of view. Therefore, the probability of the radiative recombination of the photo-excited charge carriers in the smaller NPs is considerably reduced. Consequently, large NPs become optically active and give their contribution in the PL spectrum, resulting in the observed red shift. This mechanism explains the high PL peak variation found in squalane (−0.91 meV/K). Indeed, from 303 to 383 K, the dynamic viscosity of squalane decreases by a 7.5 factor, from 22.6 to 3 mPa.s.

**Figure 5:**
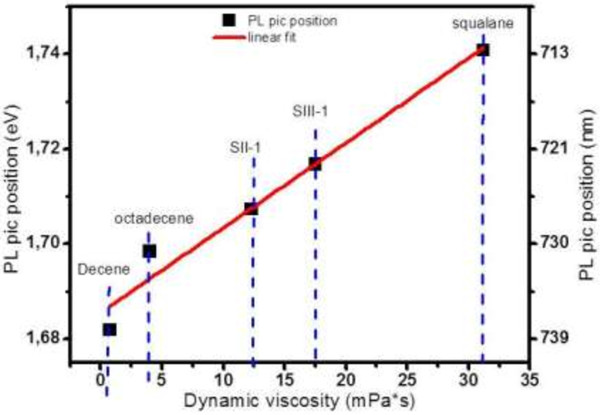
PL peak position evolution as a function of dynamic viscosity for different liquids at 300 K.

In order to assess this mechanism, we have measured the PL peak position as a function of liquid viscosity. Alkyl-capped Si NPs dispersed in five different liquids (decene, octadecene, SII_1 (mixture of octadecene and squalane with volume ratio of 0.45 and 0.55, respectively), SIII_1 (mixture of octadecene and squalane with volume ratio of 0.26 and 0.74, respectively), and squalane) with a concentration of 1 mg/mL were prepared. The dynamic viscosities of the liquids are respectively 0.73, 4, 12.3, 17.5, and 31.2 mPa.s at 25°C. Figure 5 shows the evolution of the PL peak position as a function of the dynamic viscosity of the liquids at 300 K. We clearly observe an almost linear red shift of 60 meV from squalane to decene.

Thus, a question arises: does the observed red shift of the PL peak position with temperature result from a variation of viscosity or from the intrinsic properties of the Si NPs (thermal dilation, electron-phonon interaction)?

To answer this question, we looked for a point of same viscosity for two different liquids: at 368 K, the dynamic viscosity of squalane (3.6 mPa.s) is equal to the dynamic viscosity of octadecene at 303 K. The PL peak position of Si NPs is equal to 1.702 eV in octadecene at 303 K and is equal to 1.68 eV in squalane at 368 K. Therefore, there is a difference of 22 meV between the two PL peak positions which is very close to the shift given by the Varshni expression on bulk Si (17.5 meV) in the same temperature range (from 303 down to 368 K). Hence, when corrected from the viscosity effect, the red shift that we observed (around −0.3 meV/K) with temperature is close to the one reported by different groups.

## Conclusion

Si NPs prepared by electrochemical etching of bulk Si have been functionalized with alkyl chains (octadecene) for dispersion in NPLs like lubricants for mechanical bearings. Their potential application as fluorescent nanosensors for temperature measurement in lubricated contact with optical access has been evaluated. The important variation of the fluorescence emission energy with temperature (−0.9 meV/K) allows simple temperature measurement in squalane. Nevertheless, we have shown that this variation is mainly due to energy exchange between Si NPs promoted by viscosity reduction when the temperature is increased. For static condition in the fluid, this indirect temperature sensing via viscosity change is convenient, but in dynamic conditions of the mechanical contact, a more intrinsic measurement like PL lifetime [21] is needed.

## Abbreviations

DLS: dynamic light scattering; EET: exchange energy transfer; EtOH: ethanol; FRET: Förster resonant energy transfer; FS: free-standing; FTIR: Fourier transform infrared spectroscopy; HF: hydrofluoric acid; ME: matrix-embedded; NPs: nanoparticles; NPLs: nonpolar liquids; PDMS: polydimethylsiloxane; PL: photoluminescence; QDs: quantum dots; Si: silicon; TEM: transmission electron microscopy.

## Competing interests

The authors declare that they have no competing interests.

## Authors’ contributions

HH wrote the manuscript and carried out the experiments and data analysis. DP, PV, GG, MQ, GB, and JMB guided the experiment's progress and manuscript writing and participated in mechanism discussions. SA, NPB, VM, and YC helped measure and collect the experimental data. All authors read and approved the final manuscript.

## Authors’ information

HH has obtained his Master's degree in Physics and Materials in June 2011 at University of Poitiers (France). In October 2011, he started his current Ph.D. project at Lyon Institute of Nanotechnologies. His main scientific interest focuses on synthesis, chemical functionalization, and optical characterization of silicon-based semiconductor nanostructures. SAA received his Master's degree in Chemistry from Kiev National Taras Shevchenko University in 1998 and then his Ph.D. degree in Chemistry at the same university in 2003 for his work on the ‘Immobilization of organic acids on silica gel surface, thermochemical and catalytic properties of materials obtained’. Currently, SAA is working as an associate professor in the Chemistry Faculty of the same university. Since 2004, SAA has close scientific collaboration with INSA Lyon (France); he participated in European projects such as INTAS, IRSES, and LST. Fields of his research interests are as follows: surface chemistry of nanostructured materials (semiconductors, inorganic oxides), surface functionalization and characterization, and application of nanostructures in LDI mass spectrometry, sensors, and catalysis. GG received his Master's degree in Solid State Physics from Claude Bernard University in Lyon (France) in 1970 and then his Ph.D. degree in the same field and university in 1976 for his work on the ‘Creation of defects in alkaly halides at low temperatures’. Currently GG is Professor Emeritus at INSA Lyon. From 1996 to 2007, he was the head of LPM (Laboratory of Physics of Materials) and then the vice head of INL (Institute of Nanotechnology of Lyon) from 2007 to 2010. He participated in seven European projects in the field of Materials and Devices for Microoptoelectronic. Fields of his research interests are as follows: defects in semiconductor materials and devices, and characterization of semiconductor nanostructures. NB studied at the University of Liverpool: MPhys in 2000 and Ph.D. Surface Physics in 2004. His Ph.D. was under the supervision of Prof. Peter Weightman and involved the study of ultrathin metallic surface alloys. NB has worked as a postdoctoral fellow at the University of Surrey, the Claude Bernard University Lyon, and Ecole Centrale Lyon with a common theme of physical characterization of nanomaterials. Since 2010, NB is a research engineer at the Claude Bernard University Lyon specializing in electron microscopy applied to the study of nanomaterials. VM received her Ph.D. degree in Physics from Université Joseph Fourier in Grenoble (France) in 2006. She worked on the elaboration of organic nanocrystals in sol-gel films for sensing applications. Between 2006 and 2007, she worked as a postdoctoral researcher at Commissariat à l'Energie Atomique (CEA) in Grenoble on the synthesis of FePt nanoparticles for magnetic data storage. Since 2008, she has been working as an assistant professor at Ecole Centrale de Lyon (France). Her research interest focuses on colloid (metal and oxide) synthesis and optical properties of hybrid nanoparticles working with plasmonic/fluorescent coupling. YC received his Ph.D. degree in Material Science from the Ecole Polytechnique Fédérale de Lausanne at Lausanne (Switzerland) in 1999. Between 1999 and 2001, he worked as an assistant of Pr Mathieu at EPFL on bacterial adhesion to PVC endotracheal tubes and was in charge of ToF-SIMS analysis. From 2001 to 2004, he worked in the research department of Goemar SA, Saint Malo (France). Since 2004, he joined the CNRS as a senior scientist. He focuses on microfabrication, surface chemistry and characterization, and biochips in particular glycoarrays. GB is a full Professor of the University in physics, optoelectronic, electronic, optronic and systems at INSA (Applied Sciences National Institute) of Lyon since 2001 and makes his research to the Institute of the Nanotechnology of Lyon where he was responsible of the development of new tools for nanocharacterization. He had put in place and coordinated a platform of nanoscopy since 2001. During his Ph.D. (1981) and ‘Doctorat d'Etat’ (1988), he has developed electro-optical spectroscopy techniques for the study of the physics of the deep level centers in the compound semiconductors. As a researcher of the CNRS from 1983 till 2001, he contributes to the development of InP and, afterwards (1994 to 2000), of the semiconducting photorefractive materials (InP:Fe, CdTe:V). Between 1991 and 1998, he studied the optical and electronic properties of heterostructures SiGe/Si and contributed to their integrations in devices for microelectronics (TBH, MOSFET) and for optoelectronics (photodetector, photovoltaic). He was the head of the group ‘Matériaux et Composants Micro-Optoélectronique’ of the ‘Laboratoire de Physique de la Matière’ at INSA Lyon where he studied the electronic and optical properties of Ge/Si nanostructures or InAs/InP quantum dots or Si nanocrystals in dielectrics. Since 2001, as coordinator of a platform of nanoscopy he put in place, he developed electric measurements by atomic force microscopy (AFM) with conductive tips to sound the local electronic properties of nanostructures of semiconductors with strong application potentiality. Since 2003, he is involved in the study of the third-generation high-efficiency photovoltaic cells where he has coordinated an ANR-PV project in 2006. He is a member of the team ‘Spectroscopie et nanomatériaux’ of the INL. Its whole research activity gave rise to more than 200 publications in scientific journals and in symposium proceedings. MQ finished his career in 2013 at LaMCoS, in the Group of Models Lubrication and Lubricants (ML2). His activities include the study of fluid lubrication mechanisms using physical methods (optical, Raman and fluorescence) and the consideration of liquid free surface and wetting phenomena. DP obtained his Ph.D. degree in 2007 at Ecole Centrale de Lyon (France) in the field of Tribology and Materials Science. After a postdoctoral position at the Institute for Material Science (Seville, Spain), he joined INSA of Lyon as an assistant professor in 2010. Currently, he is conducting his research activities in the Mechanics Laboratory Contacts and Dynamics (LaMCoS). His main scientific activity focuses on experimental studies in rheology, tribology, and elastohydrodynamic lubrication. PV graduated from INSA Lyon where he defended a Ph.D. in Mechanical Engineering in 1985. In 2002, he got a CNRS position as a senior scientist (Directeur de Recherche). His scientific current interests are focused on (i) the rheological and tribological behavior of multiphase or complex fluids under severe conditions, (ii) the development of multiphysics and multiscale models (by FE, FSI, MD methods) in the context of thin film lubrication, and (iii) the in situ techniques (i.e., colorimetric interferometry, Raman microspectrometry, and nanoparticle fluorescence) that make it possible to map physical parameters within highly confined thin films. Since May 2013, PV is the academic holder of the SKF research chair on ‘Lubricated interfaces for the future’ funded by SKF, a world leader company in rolling bearing manufacturing. JMB obtained his Ph.D. degree in 1996 at the University of Montpellier in the field of Condensed Matter. After two postdoctoral positions in Grenoble, he joined INSA of Lyon as an associate professor in 1999. Currently, he is conducting his research activities in the Lyon Institute of Nanotechnology. His main scientific activity focuses on spectroscopic characterizations of semiconductor nanostructures.
